# Professional Quality of Life in Research Involving Laboratory Animals

**DOI:** 10.3390/ani11092639

**Published:** 2021-09-08

**Authors:** Olatz Goñi-Balentziaga, Sergi Vila, Iván Ortega-Saez, Oscar Vegas, Garikoitz Azkona

**Affiliations:** 1 Department of Clinical and Health Psychology and Research Methodology, Euskal Herriko Unibertsitatea (UPV/EHU), Tolosa Hiribidea, 20018 Donostia, Spain; olatz.goni@ehu.eus; 2 Scientific and Technological Centers (CCIT), University of Barcelona (UB), Lluis Solé I Sabarís, 1-3, 08028 Barcelona, Spain; sergivilab@ub.edu (S.V.); ivanortegasaez@ub.edu (I.O.-S.); 3 Department of Basic Psychological Processes and Their Development, Euskal Herriko Unibertsitatea (UPV/EHU), Tolosa Hiribidea, 20018 Donostia, Spain; o.vegas@ehu.eus

**Keywords:** wellbeing, workplace, professional quality of life, compassion satisfaction, compassion fatigue, laboratory animal

## Abstract

**Simple Summary:**

Medical research involving human subjects requires previous results obtained from animal experimentation. A team of animal caretakers, technicians, welfare officers and veterinarians (animal-facility personnel) provide the husbandry and care of animals at many institutions. Researchers, on the other hand, interact with animals only when a specific procedure is being conducted. Working with laboratory animals can bring satisfaction but it can also result in workplace stress. In this study we aimed to investigate the work-related quality of life of biomedical research professionals working with laboratory animals in Spain. Animal-facility personnel showed higher professional-quality-of-life and compassion-satisfaction scores than researchers. Perceived animal stress/pain, human–animal interaction and social support are other factors that influence professional quality of life. By job category, welfare officers/veterinarians and principal investigators reported the highest scores, whereas Ph.D. students reported the lowest, indicating that job category is a contributing factor in professional quality of life. Our study may help in designing future studies or interventions to improve workplace wellbeing of the Spanish population working with laboratory animals.

**Abstract:**

Many workers contribute to the success of animal welfare and study outcomes in biomedical research. However, the professional quality of life (ProQoL) of those who work with laboratory animals has not been explored in Spain. To this end, we adapted the ProQoL scale to the Spanish population working with laboratory animals. Participants were contacted by email and asked to complete an anonymous on-line questionnaire. The study comprised a total of 498 participants, 12.4% welfare officers/veterinarians, 19.5% caretaker/technicians, 13.9% principal investigators, 20.7% investigators, 13.6% research technicians, and 19.9% PhD students. The adapted scale revealed very good reliability and internal validity, providing information about two different subscales, compassion satisfaction and compassion fatigue. Animal-facility personnel showed higher total ProQoL and compassion-satisfaction scores than researchers; PhD students showed the lowest scores. Thus, our results indicate that job category is a contributing factor in perceived professional quality of life. We observed that compassion satisfaction is negatively associated with the perceived animal stress/pain. Participants reporting poorer compassion satisfaction also reported lower social-support scores. Overall, our ProQoL scale is a useful tool for analyzing the professional quality of life in the Spanish population, and may help to design future interventions to improve workplace wellbeing in Spain and other Spanish-speaking populations.

## 1. Introduction

The Declaration of Helsinki (DH; 1964–2013) is recognized as one of the most authoritative statements on the ethical standards for human research in the world [1]. This code points out that medical research involving human subjects must be based on previous results obtained in animal experimentation. Laboratory animals provide a complex biological system in which we can control and standardize their genetic and environment, intervening and sampling when it is needed. The replacement–reduction–refinement (3Rs) principles are a framework that promotes the replacement of animals for non-sentient alternatives, the reduction of animal use, and the refinement of experiments and husbandry conditions to cause minimum pain and distress [2].

Welfare of non-human animals is an issue of great importance for European citizens [3], but the level of concern is different among European countries, the animal species involved, and their specific use [4]. Experimentation using mice is acceptable for the 66% of Europeans if it leads to an improvement in human health and wellbeing, but only 44% agree (and 37% disagree) to the use of dogs or monkeys for the same purpose [5]. In 2018, within the Member States of the European Union and Norway, rodents (52.1% mice and 9.5% rats) were the most used species, whereas the use of cats, dogs, and non-human primates represented 0.3% of total animals used for scientific purposes [6].

The human interaction with laboratory animals is an important factor in animal experimentation. It has been shown that gentle handling improves animal welfare, allows for less challenging behavioral testing, better data collection and reproducibility [7,8]. In the same way, the relationship between laboratory animals and those who work with them has an overwhelming impact on emotional health. This emotional impact is exacerbated by the responsibilities of working with other sentient beings and how best to ensure their wellbeing following interventions that can cause certain degrees of harm or distress [9].

A team of animal caretakers, technicians, welfare officers and veterinarians (i.e., animal-facility personnel) provide the husbandry and care of the animals at many institutions and contribute to their wellbeing and the success of research outcomes. Researchers usually interact with animals only when a specific procedure is being conducted [10]. Working with laboratory animals can bring satisfaction but it can also result in workplace stress [11]. Thus, on one hand, people can feel compassion satisfaction (CS), known as the pleasure that can be derived from an individual’s ability to perform work well, contribute to the work setting and the greater good of society [12]. It has been described that working closely with research animals can increase perceived CS due to the strong human–animal bonds that may develop [10]. On the other hand, people working and caring for animals can experience compassion fatigue (CF) defined as “a psychological syndrome, comprised of occupational-induced traumatic stress, and burnout, which can adversely affect those who work in caring professions” [12]. CF in animal-care professionals may lead to a reduced quality of life and is associated with loss of empathy, isolation, dissociation, substance abuse, physical ailments, trouble sleeping, and feelings of anger and sadness. CF also has negative implications from an institutional and economic standpoint, as it is associated with increased absenteeism, higher worker compensation costs, employee turnover, greater friction between staff and management, and reduced ability of personnel to complete tasks and meet deadlines. All of these things may contribute to economic losses for the institution and may indirectly jeopardize the quality of care provided to research animals [13]. In recent years, there has been an increased recognition that people working with laboratory animals are potentially vulnerable to moral stressors innate to work with animals in research [14]. Moral stress refers to having to act in a way that is different from what someone feels is ethically correct. In research settings, animal euthanasia is an area of moral stress [11], and it is thought to be one of the causes of workplace stress [15]. Euthanasia can also induce emotional dissonance; a conflict between experienced emotions and expressed emotions. Workers may feel simultaneously negative emotions from performing stressful tasks or euthanasia, but also feel unable or unsupported in expressing these emotions [16]. The need to euthanize animals after a study may lead to a “caring–killing paradox” situation, in which people that has been taking care of animals have to euthanize them [17]. This paradox may be exacerbated when there is a stronger attachment due to more frequent or intense interaction, or for animals with closer evolutionary relationship to humans [16]. It has been shown that personnel working with laboratory animals may also perform or view procedures that cause pain and distress during an experimental procedure, which alone could lead to occupational or perpetration-induced traumatic stress [15,18]. The lack of social support at work and/or home is another factor that could induce workplace stress [15]. Working in an animal laboratory may lead to social isolation because of concerns about negative social views or public pressure, the secrecy and confidentiality that some organizations encourage, lack of support from their fellows or the requirement to work unsocial hours for some studies [16,18,19].

In order to minimize the potential cost of caring on the mental wellbeing of personal working with laboratory animals, different approaches have been described to ensure a positive culture of care in research animal facilities [8,11]. However, to our knowledge, only two studies conducted in North America have analyzed animal-facility personnel mental wellbeing [13,19]. Participants in both studies reported experiencing feelings of CF. In addition, animal-facility personnel that reported higher CF also reported lower social support. On the other hand, higher CS was associated with higher social support, less animal stress/pain and more human–animal interaction. Control over euthanasia was another factor associated with the professional quality of life of laboratory animal personnel [19].

In our previous work we observed that people working with laboratory rodents in biomedical research in Spain reported that, in their opinion, animals suffer low levels of stress and pain. A sizable proportion of researchers and animal facility caretakers and technicians stated that they did not have control over euthanasia (were never given the choice not to euthanize the rodents they work with). Researchers showed the lowest human–animal interactions, but they also spent less hours working directly with the animals. Welfare officers and/or veterinarians showed the highest scores in social support, although the vast majority of participants reported that they tend not to talk about their work with people outside their inner social circle [20]. In this study, we aimed to examine the professional quality of life (ProQoL) of animal-facility personnel and researchers working with laboratory mammals, from non-human primates to mice, in biomedical research in Spain. Thus, we adapted the ProQoL scale to the Spanish system. In addition, based on our previous questionnaire, participants were asked about their perceived animal stress/pain, euthanasia, human–animal interaction and social support. These data may contribute to determine the workplace quality of our participants but also to identify factors that may help us to design future interventions to increase CS and reduce CF.

## 2. Materials and Methods

### 2.1. Participants and Procedure

Participants were recruited by e-mail trough the e-mail list of the Spanish Society for Laboratory Animal Science (SECAL-L) and direct e-mails to known laboratory personnel between 1 December 2020 and 31 May 2021. This study was restricted to people working with laboratory animals (mammals) in Spain. In a cover letter attached to the questionnaire, participants were informed that the survey data would be used for scientific purposes and that they would remain anonymous. All participants gave their voluntary informed consent prior to completing the short 10-min online questionnaire (Google Drive platform).

### 2.2. Instruments

The survey contained questions related to participant’s gender, age, education, current professional role, institution, hours working directly with laboratory animals per week, and total years.

We adapted a professional quality of life (ProQoL) scale [12,19] in order to measure the perceived work-related quality of life in relation to their work with laboratory animals during the 30 days. ProQoL scale comprises two major aspects: the positive (compassion satisfaction, CS) and the negative (compassion fatigue, CF). We translated the ProQoL scale into Spanish following a forward-backward design [21]. Each item was translated into Spanish by two bilingual researchers and then they were compared and discussed until a consensus was reached regarding the wording of each item. Two different bilingual researchers did back-translation and they compared it until they reached a consensus. This translation was examined and compared with the original wording to determine whether the items had the same meaning.

Questions related to animal stress and pain, euthanasia, human–animal interaction and social support were based on our previously published work [20]. Participants were asked to self-assess the degree of stress and pain of the animals they work with, using categories based on Spanish legislation (RD53/2013); *little to none*, *minor*, *moderate*, or *severe*. Euthanasia practices were assessed by asking participants, firstly, whether they had ever euthanized a laboratory animal; *yes* or *no*. Participants that responded yes were asked about the frequency they do it: *less than a month*, *monthly*, *weekly* or *daily*, and, after, to respond to the statement *“I get to decide whether I am the one to euthanize the animals I have cared for“* with one of the following options: *never*, *sometimes*, *about half of the time*, *most of the time* or *always*. Human–animal-interaction score was assessed by asking participants how strongly they agreed or disagreed, from one (*strongly disagree*) to seven (*strongly agree*) about how often they observed, pet, talked to or named the laboratory animals. Thus, the maximum score that a participant could obtain was 28. Social-support score was assessed trough questions directed to rate from one (*never*) to five (*always*) how often they talked to friends and/or family and to people outside their social circle about their work with laboratory animals, and how often they felt they had someone they could really count on when dealing with stress related to their work. The maximum score that a participant could obtain was 15.

### 2.3. Statistical Analysis of Data

All statistical analysis were performed with Jamovi (1.16.15) and FACTOR (10.03.01) software. The level of significance was set to *p* < 0.05. Frequency (%) and distribution—mean ± standard deviation (SD)—statistics were used to describe the sample. Means, SD and homogeneity indexes were computed for each ProQoL item. ProQoL total-score and subscales’ reliability were analyzed using standardized Cronbach’s alpha and McDonald’s ordinal omega coefficients [22]. Since we adapted the items to professionals working with laboratory animals, we carried out an exploratory factor analysis instead of a confirmatory factor analysis. The number of factors were determined by the unweighted least squares (ULS) for ordinal variables using a parallel analysis procedure. This analysis provides parameter estimates in the model, along with several goodness-of-fit measures, which include the chi-square test, the goodness-of-fit index (GFI), and the comparative-fit index (CFI). For all three indices, values in the mid 0.90 range and above are indicative of optimal fit. The root-mean-square error of approximation (RMSEA), root-mean-square error of residual (RMSR), and weighted root mean square residual (WRMR) are other fit indeces used; values under 0.05, 0.08 and 1, respectively, are indicative of a good model fit [23]. Mann–Whitney U test (*U*) was used to compare animal-facility personnel and researchers’ scores, and Kruskal–Wallis one-way analysis of variance followed by Mann–Whitney U test for post-hoc analysis for the six job categories and gender. Rank biserial correlation was used to analyze the effect size. Relation between ProQoL scores and demographic information, stress/pain, euthanasia, human–animal interaction and social support were analyzed using bivariate Spearman correlation. There was no missing data.

## 3. Results

### 3.1. Demographic Information

The study comprised a total of 498 participants, of which 335 were cis/trans women (67.3%), 151 cis/trans men (30.3%) and 12 preferred not to say (2.4%) (Table 1). Their ages ranged from 21 to 69 years (36 ± 10.4). Two participants finished primary school (0.4%), 19 secondary school (3.8%), 71 hold a vocational training degree (14.3%), 216 a Bachelor degree (43.4%) and 190 a Ph.D. (38.1%). Animal-facility personnel were divided into two groups; welfare officers and/or veterinarians (62/12.4%) and animal caretaker or technicians (97/19.5%). Researchers were divided into four groups: principal investigators (69/13.9%), investigators (103/20.7%), research technicians (68/13.6%), and Ph.D. students (99/19.9%). They worked in research institutes (236/47.4%), universities (154/30.9%), hospitals (47/9.4%), contract research organizations (42/8.4%), or pharmaceutical companies (19/3.8%). Participants reported experience from less than a year to a maximum of 43 years (10 ± 8.96) working with laboratory animals. Animal-facility personnel reported working 28 h ± 14 per week and researchers 10 h ± 10 per week. The vast majority of participants worked with rodents, mice (359/72.1%) and rats (158/31.7%), but some participants also reported working with pigs (94/18.9%), dogs (22/4.4%) or non-human primates (11/2.2%), among other species that are summarized in Table S1.

### 3.2. ProQoL Scale’s Psychometric Properties

For computation, we first reversed all the inverse items. Then, we computed means and standard deviations of the 30 items. The value of Kaiser–Meyer–Olkin (KMO = 0.863) and Bartlett’s test (Bartlett’s statistic_(435)_ = 5597.2; *p* < 0.001) obtained indicated that the correlation matrix was adequate for the factor analysis [24]. The exploratory factor analysis classified the items in two subscales: 1) compassion satisfaction (CS), and 2) compassion fatigue (CF). The goodness-of-fit statistics (X^2^_(376)_ = 876.198; *p* < 0.001/GFI = 0.981/CFI = 0.984/RMSEA = 0.052/RMSR = 0.058/WRMR = 0.061) indicated a good model fit, and the factors explained a large percentage of variance (CS = 17.94% and CF = 38.58%). A positive relation was observed between factors (*r* = 0.441). The reliability analysis showed a very good internal consistency for all the tests (α = 0.93/ω = 0.93) and each of the two subscales (CS subscale α = 0.92/ω = 0.92, and CF subscale α = 0.93/ω = 0.93). However, we could not analyze separately burnout and secondary traumatic stress within CF subscale. The mean, SD and homogeneity indices for each item are described in Table S2 in Spanish and Table S3 in English. The exploratory factor analysis indicated that item 25 had to be in both subscales, whereas item 26 could only be included in the total score, but not in the subscales.

### 3.3. Professional Quality of Life

Scale and subscale score analyses did not show any difference in relation to gender. Even though statistically significant, the observed correlations were negligible or weak between ProQoL scores and age (total score: rho = 0.199; *p* < 0.001/CS: rho = 0.185; *p* < 0.001/CF: rho = 0.147; *p* < 0.001); years working with laboratory animals (total score: rho = 0.177; *p* < 0.001/CS: rho = 0.166; *p* < 0.001/CF: rho = 0.129; *p* = 0.004), and hours worked per week (total score: rho = 0.126; *p* = 0.005/CS: rho = 0.295; *p* < 0.001/CF: rho = −0.122; *p* = 0.023).

**Figure 1:**
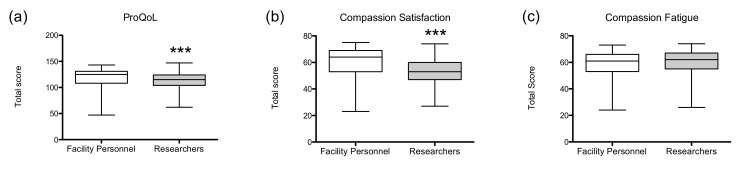
Animal-facility personnel versus researchers (**a**) professional quality of life scale (max 150), (**b**) compassion satisfaction (max 75) and (**c**) compassion fatigue (max 75) subscale scores. Data are presented as group median (min to max). *** *p* < 0.001.

We first compared ProQoL scores between animal-facility personnel and researchers. Facility personnel showed significantly higher total ProQoL scores than researchers (U = 19953; *p* < 0.001; *r* = 0.260; Figure 1a). The same results were obtained in the CS subscale (U = 15402; *p* < 0.001; *r* = 0.429; Figure 1b), but not in the CF subscale (U = 24581; *p* = 0.113; *r* = 0.088; Figure 1c).

We next explored, in depth, the reported scores, considering the six job categories mentioned above. Overall, our analysis revealed significant differences in total ProQoL score (*X*^2^ _(__5)_ = 59.9; *p* < 0.001), CS (*X^2^* _(5)_ = 80.6; *p* < 0.001) and CF (*X*^2^ _((5)_ = 38.9; *p* < 0.001). The results of the post-hoc analysis are summarized in Table 2. Briefly, principal investigators obtained the highest ProQoL score, which was statistically significant compared with investigators (*U* = 2263; *p* < 0.001; *r* = 0.363), research technicians (*U* = 1440; *p* < 0.001; *r* = 0.386), and PhD students (*U* = 1468; *p* < 0.001; *r* = 0.570). In the same way, welfare officers and/or veterinarians reported significantly higher total scores than investigators (*U* = 2362; *p* = 0.005; *r* = 0.260), research technicians (*U* = 1483; *p* = 0.004; *r* = 0.296), and PhD students (*U* = 1677; *p* < 0.001; *r* = 0.454). Compassion satisfaction (CS) subscale scores indicated that welfare officers and/or veterinarians reported higher CS than all research job categories; principal investigators (*U* = 1626; *p* = 0.018; *r* = 0.240), investigators (*U* = 1766; *p* < 0.001; *r* = 0.447), research technicians (*U* = 1194; *p* < 0.001; *r* = 0.434) and PhD students (*U* = 1215; *p* < 0.001; *r* = 0.604). At the other end, PhD students reported the lowest CS score with significant differences compared with the rest of the job categories; principal investigators (*U* = 1888; *p* < 0.001; *r* = 0.447), investigators (*U* = 4148; *p* = 0.022; *r* = 0.187), research technicians (*U* = 2646; *p* = 0.019; *r* = 0.214), and animal caretaker/technicians (*U* = 2136; *p* < 0.001; *r* = 0.555). Principal investigators showed the highest compassion-fatigue (CF) score, meaning that they reported low levels of CF. This group reported lower fatigue than that obtained by welfare officers and/or veterinarians (*U* = 1218; *p* < 0.001; *r* = 0.431), caretakers/technicians (*U* = 1848; *p* < 0.001; *r* = 0.448), investigators (*U* = 2251; *p* < 0.001; *r* = 0.367), research technicians (*U* = 1563; *p* < 0.001; *r* = 0.334), and PhD students (*U* = 1643; *p* < 0.001; *r* = 0.519). Results indicated that PhD students’ CF score was significantly lower than the score obtained for investigators (*U* = 4003; *p* = 0.008; *r* = 0.215). No statistical differences were observed between welfare officer and/or veterinarians and caretakers/technicians.

### 3.4. Animal Stress/Pain and Euthanasia

The analysis of variance indicated significant differences in the perceived animal stress/pain levels by job category (X^2^_(5)_ = 41.6; *p* < 0.001). PhD students reported the highest levels (4.95 ± 1.3) and animal caretakers/technicians the lowest (3.88 ± 1.3). A Spearman correlation indicated a negative moderate correlation between perceived animal stress/pain and ProQoL total score (rho = −0.317; *p* < 0.001), and the CS subscale (rho = −0.336; *p* < 0.001) and a negligible correlation with the CF subscale (rho = −0.174; *p* < 0.001). Regarding euthanasia, our results indicated that killing animals was not a factor that affecte the professional quality of life (U = 5806; *p* = 0.879; *r* = 0.018). Euthanasia frequency was neither correlated with ProQoL score, even if we observed significant differences by job category (X^2^_(5)_ = 40.9; *p* < 0.001). Finally, regarding control over euthanasia, we had two clearly separated groups, welfare officers/veterinarians, principal investigators and investigators reported the highest control over euthanasia, whereas animal caretakers/technicians, research technicians and PhD students the lowest (X^2^_(5)_ = 50.3; *p* < 0.001). In this case, a positive low correlation was observed with ProQoL total scale (rho = 0.204; *p* < 0.001), but negligible correlation with the CS subscale (rho = 0.159; *p* < 0.001) and the CF subscale (rho = 0.164; *p* < 0.001).

### 3.5. Perceived Interaction with Animals and Social Support

**Figure 2:**
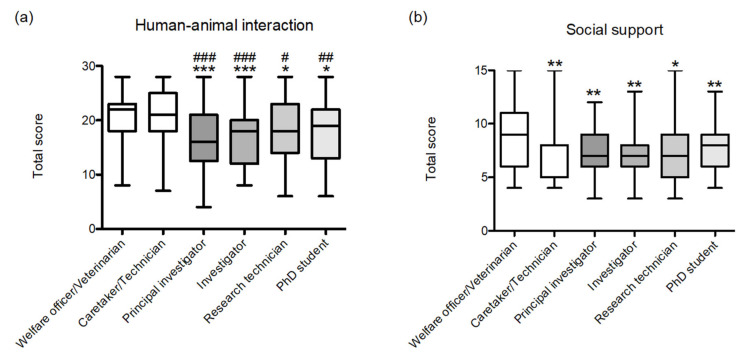
Total score of (**a**) human–animal interaction (max 28) and (**b**) social support by job category (max 15). Data are presented as group median (min to max). * *p* < 0.005, ** *p* < 0.01, *** *p* < 0.001 versus welfare officers and/or veterinarians; ^#^ *p* < 0.05, ^##^ *p* < 0.01, ^###^ *p* < 0.001 versus animal-facility caretakers or technicians.

According to the analysis of variance, significant differences were observed in the total human–animal interaction score by job category (*X*^2^_(5)_ = 45.2; *p* < 0.001). Overall, animal-facility personnel obtained higher scores than researchers (Figure 2a). A Spearman correlation indicated no correlation between human–animal interaction and ProQoL total score (*rho* = 0.080; *p* = 0.075), and only a positive low correlation with the CS subscale (*rho* = 0.283; *p* < 0.001) and a negative low correlation with the CF subscale (*rho* = −0.221; *p* < 0.001).

Analysis of social-support scores indicated significant differences according to job category (*X*^2^_(5)_ = 22.8; *p* < 0.001). Specifically, welfare officers and/or veterinarians reported higher levels of social support than the other five job categories (Figure 2b). Positive correlations were found with ProQoL total score (*rho* = 0.291; *p* < 0.001), the CS subscale (*rho* = 0.307; *p* < 0.001) and the CF subscale (*rho* = 0.161; *p* < 0.001).

## 4. Discussion

In our study we aimed to analyze the professional quality of life of people working with laboratory animals in biomedical research in Spain. To this end, we first adapted the ProQoL scale for Spanish population working with laboratory animals. In general, although a few items did not show good homogeneity-index values, the adapted scale revealed very good reliability and internal-validity parameters, providing information about two different dimensions, compassion satisfaction (CS) and compassion fatigue (CF). However, in the adaptation, we lost some sensibility, since we could not evaluate, separately, burnout and secondary traumatic stress within the CF subscale.

As in previous studies [13,19,20], our participants were mostly women, but our results showed that gender was not a factor that influenced scores of professional quality of life. In the same way, age, and the number of years and hours working directly with laboratory animals minimally affected it. Unfortunately, we could not study the effect of working with animals that are phylogenetically or emotionally closer to human beings, though many participants reported working with several animal species.

Our results indicated that people who made the decision to work in the field of laboratory-animal welfare (animal-facility personnel) showed higher ProQoL and CS than researchers. Animal-facility staff also reported the highest human–animal interaction scores, and, although weak, we observed a positive correlation with CS. This correlation is in line with a previous work [19], and may indicate that animal-facility personnel may take more satisfaction from assuring laboratory animals’ wellbeing and having a closer relationship with them, because they do not usually perform the procedures that cause their suffering.

On the other hand, no differences were observed between animal-facility personnel and researchers in the CF subscale. This is a topic of increasing concern because it can affect the mental wellbeing of people caring for animals and can adversely impact their quality of life [18,25]. In fact, CF has been described in laboratory-animal personnel in Canada and the USA [13]. Since participants in our study were recruited via convenience sampling, two main limitations should be noted. First, individuals with severe CF may be less likely to participate because they may be withdrawing from any additional responsibilities related to their job and, therefore, we could have missed data from them. Second, individuals who may have previously worked with laboratory animals but left their positions due to CF are also absent from the study.

Our results indicate that job category influences professional quality of life. Those participants in senior positions reported the highest professional quality of life (principal investigators the highest total ProQoL and CF scores and welfare officers/veterinarians the highest CS score). These results suggest that having control over the management of animals and years of experience positively working with them influence the professional quality of life. In agreement with this idea, PhD students reported the lowest scores on all scales. This group also reported the highest perceived animal stress/pain levels, which also showed a negative correlation with professional quality of life. This result may not be related solely to their work with laboratory animals, since different studies reported high levels of burnout and a poor work–life balance among biomedical PhD students worldwide [26,27] and in Spain [28]. Moreover, this poorest quality of life can be a risk factor for developing common psychiatric disorders, especially clinical depression [29].

Euthanizing animals is thought to be one of the causes of workplace stress for many animal-care workers [15]. However, we did not observe a difference between participants that had or had not euthanized animals. Moreover, although it has been reported that the control over performing euthanasia is related to professional quality of life [19], our results showed only a weak positive correlation. In line previous reported [19], we did not observe a correlation between euthanasia frequency and ProQoL scale. The cultural difference in the perception about the euthanasia can explain the discrepancies between studies. Unfortunately, we were not able to analyze the influence of the method because the participants in our study reported using more than one.

Social-support networks are considered key to minimizing workplace stress [15] and so maintaining quality of life [30,31]. As previously reported [19], our results indicated that social support correlates positively with professional quality of life. By job category, welfare officers/veterinarians reported the higher score. Importantly, different studies have shown that people working with laboratory animals may have difficulty gaining work-related social support because of the stigmatization of the field [19,32]. In our previous work [20], we observed that people working with laboratory rodents are cautious and rarely talked about their job, suggesting that it is considered a sensitive issue in Spain. In future studies, it would be interesting to delve into the study effect of this taboo, encompassing other aspects of social support, in order to have a broader vision and to better understand inside- and outside-of-work influences, which could be modified to improve professional quality of life for such workers.

Although we have identified certain limitations, as previously discussed, our adapted ProQoL scale will allow future studies to identify other variables that may influence the professional quality of life (e.g., the use of environmental enrichment, empathy levels, etc.). In addition, the scale permits studying the efficacy of an intervention designed to improve the professional quality of life, which, according to our data, should be focused on reducing the stress and pain of animals, increasing control over euthanasia, increasing interaction with animals, and improving social support. Particularly, we have identified that PhD students represent a vulnerable group and special policies should be implemented to prevent burnout in these first steps of their research career.

## 5. Conclusions

Our study indicates that animal-facility personnel show higher perceived professional quality of life in research involving laboratory animals than researchers. By job category, participants in senior positions reported the highest professional quality of life, whereas PhD students reported the lowest. Thus, our results indicate that job category may be a factor that determines the professional quality of life in Spanish workers; potentially, the higher the job category, the higher the professional quality of life. Perceived animal stress/pain, control over euthanasia, human–animal interaction, and social support are likely factors associated with professional quality of life. Overall, our study provides valuable insight into the professional quality of life of people working with laboratory animals in Spain, and may help in designing future studies and evaluating interventions to improve workplace wellbeing in laboratory-animal science.

## Figures and Tables

**Table 1:** Participants gender, age and reported working years and hours-per-week by job category. Data are expressed as total numbers or mean ± SD.

Job Category		Gender			Age (Years)	Working Directly with Laboratory Animals	
		Male (Cis/Trans)	Female (Cis/Trans)	Prefer Not to Say		Years	Hours/Week
Animal-Facility Personnel	Welfare officer and/or veterinarian	19	42	1	46.1 ± 9.2	16.8 ± 8.9	22 ± 13
	Caretaker or technician	24	72	1	38.7 ± 10	10.8 ± 7.3	33 ± 13
Researchers	Principal investigator	25	42	2	46.8 ± 9.2	19.4 ± 8.9	8 ± 10
	Investigator	39	62	2	38.4 ± 8.5	12.5 ± 8.1	8 ± 8
	Research technician	16	48	4	36.3 ± 8.4	10.7 ± 8.1	16 ± 13
	PhD student	28	69	2	26.9 ± 2.1	3.2 ± 1.9	10 ± 9

**Table 2:** Professional quality of life scale and subscales scores by job category. Data are presented as group mean ± SD. ^^^^^ *p* < 0.001 versus principal investigator; * *p* < 0.05, ^**^ *p* < 0.01, *** *p* < 0.001 versus welfare officer and/or veterinarian; ^#^ *p* < 0.05, ^###^ *p* < 0.001 versus PhD students.

Job Category		Total Score	Compassion Satisfaction (CS)	Compassion Fatigue (CF)
Animal-Facility Personnel	Welfare officer and/or veterinarian	120.60 ± 14.23	61.13 ± 8.71	59.47 ± 7.93 ^^^^^
	Caretaker or technician	119.44 ± 16.92	60.08 ± 11.54 ^###^	59.36 ± 8.11 ^^^^^
Researchers	Principal investigator	122.77 ± 10.44	57.59 ± 7.92 *	65.17 ± 5.67
	Investigator	114.20 ± 13.88 **^,^^^^	53.39 ± 9.22 ***^,#^	60.82 ± 7.45 ^^^^,#^
	Research technician	111.63 ± 18.32 **^,^^^^	53.79 ± 8.96 ***^,#^	57.84 ± 12.68 ^^^^^
	Ph.D. student	106.63 ± 17.28 ***^,^^^^^^	50.03 ± 9.32 ***^,^^^^	56.60 ± 10.93 ^^^^^

## Data Availability

All the data of the study will we available upon request.

## Supplementary Materials

The following are available online at https://www.mdpi.com/article/10.3390/ani11092639/s1, Table S1: Species by alphabetic order, Table S2: Professional quality of life items in Spanish by subscales, Table S3: Professional quality of life items in English by subscales.
