# Supplementary material for: Professional Quality of Life in Research Involving Laboratory Animals
Source: Animals (Basel). 2021 Sep 8;11(9):2639. doi: 10.3390/ani11092639 (PMC8465412; doi:10.3390/ani11092639)
Supplement: Supplementary file 1 [file animals-11-02639-s001.zip › animals-1323454-supplementary.pdf]

Table S1. Species by alphabetic order

	Total	Percentage (%)
Cat *(Felis catus)*	4	0.8
Cow *(Bos taurus)*	8	1.6
Dog (*Canis lupus familiaris*)	22	4.4
Ferret *(Mustela putorius furo)*	4	0.8
Goat *(Capra aegagrus hircus)*	5	1.0
Guinea pig *(Cavia porcellus)*	9	1.8
Syrian hamster (*Mesocricetus auratus*)	6	1.2
Horse (*Equus ferus caballus*)	4	0.8
Mouse *(Mus musculus)*	359	72.1
Non-human primate	11	2.2
Pig *(Sus scrofa)*	94	18.9
Rabbit *(Oryctolagus cuniculus)*	44	8.8
Rat *(Rattus norwegicus)*	158	31.7
Sheep (*Ovis aries*)	14	2.8

Table S2. Professional quality of life items in Spanish by subscales.

ProQOL	*M*	*SD*	*r*
26. Me siento estancado/a en mi trabajo	3.26	1.18	0.32
**Compassion satisfaction (CS)**			
1. Soy feliz trabajando con animales de laboratorio	3.52	1.16	0.68
3. Me satisface poder cuidar los animales con los que trabajo	3.96	1.14	0.38
4. Me siento conectado con mi trabajo	3.89	1.08	0.69
6. Me siento revitalizado después de trabajar con los animales con los que trabajo	2.71	1.17	0.45
12. Me gusta mi trabajo con animales de laboratorio	3.73	1.15	0.70
15. Me satisface la manera en la que soy capaz de mantenerme al día con las técnicas y protocolos de supervisión	3.60	1.04	0.39
16. Soy la persona que siempre quise ser	3.57	0.90	0.60
17. Mi trabajo con animales de laboratorio me satisface	3.58	1.11	0.73
19. Tengo pensamientos y sentimientos felices hacia mis seres queridos y sobre cómo podría cuidarlos	4.14	0.90	0.19
21. Creo que puedo destacar en mi trabajo	3.64	0.87	0.39
23. Me enorgullece lo que puedo hacer para ayudar	3.96	0.89	0.56
25. Trabajar con animales de laboratorio me produce emociones negativas (R)	3.92	1.13	0.72
27. Me siento exitoso/a con mi trabajo	3.60	0.86	0.56
28. Soy una persona muy bondadosa	3.99	0.78	0.06
30. Estoy feliz de haber elegido hacer este trabajo	3.82	0.96	0.68

**Compassion Fatigue (CF)**			
2. A menudo acaricio a los animales de laboratorio con los que trabajo	2.70	1.13	0.16
5. Cualquier sonido inesperado me sobresalta (R)	3.22	1.23	0.23
7. Me resulta difícil separar mi vida personal de mi vida laboral con los animales de laboratorio (R)	3.73	1.22	0.26
8. No soy tan productivo en el trabajo porque no puedo dormir debido a experiencias traumáticas con animales de laboratorio con los que trabajé (R)	4.47	0.76	0.58
9. Creo que podría sufrir de estrés debido a un trauma originado por un animal con el que trabajé (R)	4.33	0.93	0.50
10. Me siento atrapado por mi trabajo con animales de laboratorio (R)	4.06	1.16	0.57
11. Debido a mi trabajo con los animales de laboratorio me siento agobiado en diferentes aspectos de mi vida (R)	4.26	1.00	0.67
13. Me siento deprimido debido a alguna experiencia traumática con animales de laboratorio con los que trabajo (R)	4.43	0.86	0.60
14. Siento como si estuviera experimentando un trauma debido un animal de laboratorio con el que trabajé (R)	4.65	0.74	0.56
18. Me agota mi trabajo con animales de laboratorio (R)	3.30	1.06	0.57
20. Me abruma lo interminable que parece mi carga de trabajo (R)	3.07	1.11	0.43
22. Evito ciertas actividades o situaciones porque me recuerdan a experiencias aterradoras de los animales de laboratorio con los que trabajo (R)	4.66	0.73	0.54
24. Debido a mi trabajo tengo pensamientos negativos y aterradores (R)	4.51	0.83	0.62
25. Trabajar con animales de laboratorio me produce emociones negativas (R)	3.92	1.13	0.72
29. No puedo recordar partes importantes de mi trabajo con animales traumatizados (R)	4.41	0.92	0.27

Note: (R): reverse items.

Table S3. Professional quality of life items in English by subscales.

ProQOL	*M*	*SD*	*r*
26. I feel "bogged down" by the system.	3.26	1.18	0.32
**Compassion satisfaction (CS)**			
1. I am happy working with laboratory animals	3.52	1.16	0.68
3. I get satisfaction from taking care of the animals I work with	3.96	1.14	0.38

4. I feel connected to my work	3.89	1.08	0.69
6. I feel invigorated after working with the animals I work with	2.71	1.17	0.45
12. I like my work with laboratory animals	3.73	1.15	0.70
15. I am pleased with how I am able to keep up with supervision techniques and protocols	3.60	1.04	0.39
16. I am the person I always wanted to be	3.57	0.90	0.60
17. My work with laboratory animals makes me feel satisfied	3.58	1.11	0.73
19. I have happy thoughts and feelings about my relatives and how I could help them	4.14	0.90	0.19
21 I believe I can make a difference through my work.	3.64	0.87	0.39
23. I am proud of what I can do to help.	3.96	0.89	0.56
25. As a result of my work with laboratory animals, I have intrusive, frightening thoughts (R)	3.92	1.13	0.72
27. I have thoughts that I am a "success" in my work	3.60	0.86	0.56
28. I am a very caring person.	3.99	0.78	0.06
30. I am happy that I chose to do this work	3.82	0.96	0.68
**Compassion Fatigue (CF)**			
2. I often stoke the laboratory animals I work with	2.70	1.13	0.16
5. I jump or am startled by unexpected sounds (R)	3.22	1.23	0.23
7. I find it difficult to separate my personal life from my work life with laboratory animals (R)	3.73	1.22	0.26
8. I am not as productive at work because I am losing sleep over traumatic experiences of a laboratory animal I worked with (R)	4.47	0.76	0.58
9. I think that I might have been affected by the traumatic stress of a laboratory animal I worked with (R)	4.33	0.93	0.50
10. I feel trapped by my job with laboratory animals (R)	4.06	1.16	0.57
11. Because of my work with laboratory animals, I have felt "on edge" about various aspects of my life (R)	4.26	1.00	0.67
13. I feel depressed because of the traumatic experiences of the laboratory animals I work with (R)	4.43	0.86	0.60
14. I feel as though I am experiencing the trauma of a laboratory animal I worked with (R)	4.65	0.74	0.56
18. I feel worn out because of my work with laboratory animals (R)	3.30	1.06	0.57
20. I feel overwhelmed because my work load seems endless (R)	3.07	1.11	0.43

22. I avoid certain activities or situations because they remind me of frightening experiences of laboratory animals I work with (R)	4.66	0.73	0.54
24. As a result of my work, I have intrusive, frightening thoughts (R)	4.51	0.83	0.62
25. Working with laboratory animals generated negative emotions in me (R)	3.92	1.13	0.72
29. I can't recall important parts of my work with traumatized laboratory animals (R)	4.41	0.92	0.27

Note: (R): reverse items.
